# Supplementary material for: Prevalence of Seasonal Influenza Vaccination in Chronic Obstructive Pulmonary Disease (COPD) Patients in the Balearic Islands (Spain) and Its Effect on COPD Exacerbations: A Population-Based Retrospective Cohort Study
Source: Int J Environ Res Public Health. 2020 Jun 5;17(11):4027. doi: 10.3390/ijerph17114027 (PMC7312905; doi:10.3390/ijerph17114027)
Supplement: Supplementary file 1 [file ijerph-17-04027-s001.pdf]

Table S1. Main co-morbidities in relation to Influenza Vaccination during the 2012/2013 Campaign.

	Unvaccinated		Vaccinated		Total		
	N=5003	% Row	*N*=7393	% Row	*N*=12396	% column[a]	*p value*
**High blood pressure (HBP)**							
No	2432	46.0%	2854	54.0%	5286	42.6%	<0.001
Yes	2571	36.2%	4539	63.8%	7110	57.4%	
**Diabetes**							
No	3850	43.1%	5081	56.9%	8931	72.0%	<0.001
Yes	1153	33.3%	2312	66.7%	3465	28.0%	
**Heart failure**							
No	4379	41.5%	6167	58.5%	10546	85.1%	<0.001
Yes	624	33.7%	1226	66.3%	1850	14.9%	
**Atrial fibrillation**							
No	4273	42.2%	5844	57.8%	10117	81.6%	<0.001
Yes	730	32.0%	1549	68.0%	2279	18.4%	
**Ischemic heart disease**							
No	4341	41.5%	6108	58.5%	10449	84.3%	<0.001
Yes	662	34.0%	1285	66.0%	1947	15.7%	
**Osteoporosis**							
No	4326	41.1%	6209	58.9%	10535	85.0%	<0.001
Yes	677	36.4%	1184	63.6%	1861	15.0%	
**Lung cancer**							
No	4955	40.4%	7299	59.6%	12254	98.9%	>=0.05
Yes	48	33.8%	94	66.2%	142	1.1%	
**Cor pulmonale**							
No	4942	40.5%	7274	59.5%	12216	98.5%	>=0.05
Yes	61	33.9%	119	66.1%	180	1.5%	
**Cerebrovascular Diseases**							
No	4618	41.1%	6612	58.9%	11230	90.6%	<0.001
Yes	385	33.0%	781	67.0%	1166	9.4%	
**Depressive disorder**							
No	4888	40.3%	7249	59.7%	12137	97.9%	>=0.05
Yes	115	44.4%	144	55.6%	259	2.1%	
**Anxiety disorder**							
No	3409	39.6%	5203	60.4%	86.12	69.5%	>=0.05
Yes	1594	42.1%	2190	57.9%	3784	30.5%	
**HIV**							
No	4989	40.3%	7385	59.7%	12374	99.8%	>=0.05
Yes	14	63.6%	8	36.4%	22	0.2%	
**Obstructive sleep apnea**							
No	4589	40.6%	6709	59.4%	11298	91.1%	>=0.05
Yes	414	37.7%	684	62.3%	1098	8.9%	
**Gastroesophageal reflux disease**							
No	4663	41.0%	6723	59.0%	11386	91.9%	>=0.05
Yes	340	33.7%	670	66.3%	1010	8.1%	
**Chronic kidney disease**							
No	4803	41.0%	6914	59.0%	11717	94.5%	<0.001
Yes	200	29.5%	479	70.5%	679	5.5%	
**Allergic rhinitis**							

No	4547	40.7%	6638	59.3%	11185	90.2%	>=0.05
Yes	456	37.7%	755	62.3%	1211	9.8%	

Table S2. Crude and adjusted associations between "History of Influenza Vaccination" and risk of "Admission due to COPD Exacerbation" in patients with confirmed COPD (FEV1/FVC<0.7) by obstruction severity during the complete year and the epidemic period.

	Severe Exacerbations											
	**2013 complete year**						**2013 epidemic period only**					
	**None**	**At least one**					**None**	**At least one**				
	N=3850	N=393	OR	(95% CI)	ORa	(95% CI)	N=4096	N=147	OR	(95% CI)	ORa	(95% CI)
Influenza Vaccination (N=12396)												
Unvaccinated	1464	135	1	--	1	--	1543	56	1	--	1	--
Vaccinated	2386	258	1.17	(0.94 - 1.46)	0.97	(0.74 - 1.27)	2553	91	0.98	(0.70-1.38)	0.81	(0.54-1.22)
GOLD 1 (N=1100)												
Unvaccinated	185	8	1	--	1	--	193	0	1	--	1	--
Vaccinated	263	15	1.32	(0.55 - 3.17)	0.89	(0.30 - 2.60)	274	4				
GOLD 2 (N=2995)												
Unvaccinated	730	57	1	--	1	--	759	28	1	--	1	--
Vaccinated	1199	81	0.87	(0.61 - 1.23)	0.71	(0.48 - 1.06)	1246	34	0.74	(0.45-1.23)	0.64	(0.36-1.15)
GOLD 3 (N=1333)												
Unvaccinated	352	45	1	--	1	--	378	19	1	--	1	--
Vaccinated	636	107	1.32	(0.91 - 1.91)	1.04	(0.67 - 1.62)	707	36	1.01	(0.57-1.79)	0.77	(0.40-1.48)
GOLD 4 (N=183)												
Unvaccinated	50	9	1	--	1	--	55	4	1	--	1	--
Vaccinated	81	27	1.85	(0.81-4.26)	5.82	(1.68 - 20.15)	99	9	1.25	(0.37-4.25)	2.07	0.38-11.23

OR: crude odds ratio. ORa: odds ratio adjusted for age (continuous variable), gender, concomitant asthma diagnosis, smoking status (ordinal variable: non-smoker, former smoker, current smoker), number of moderate exacerbations the previous year, number of severe exacerbations the previous year, and the following comorbities: Heart failure, Atrial fibrillation, Cor pulmonale, Anxiety disorder, Osteoporosis, Allergic rhinitis, Gastroesophageal reflux disease, and Diabetes. COPD: chronic obstructive pulmonary disease. COPD obstruction severity: GOLD 1: mild; GOLD 2 moderate; GOLD 3: severe; GOLD 4 very severe.

Table S3. Crude and adjusted associations between "History of Influenza Vaccination" and risk of moderate COPD Exacerbations" in patients with confirmed COPD (FEV1/FVC < 0.7) by obstruction severity during the complete year and the epidemic period.

Moderate Exacerbations

	**2013 complete year**						**2013 epidemic period only**					
	**None**	**At least one**					**None**	**At least one**				
	N=2144	N=2099	OR	(95% CI)	ORa	(95% CI)	N=3156	N=1087	OR	(95% CI)	ORa	(95% CI)
Influenza Vaccination (N=12396)												
Unvaccinated	876	723	1	--	1	--	1230	369	1	--	1	--
Vaccinated	1268	1376	1.32	(1.16 - 1.49)	1.14	(0.97 – 1.32)	1926	718	1.24	(1.08-1.44)	1.09	(0.92-1.30)
GOLD 1 (N=1100)												
Unvaccinated	126	67	1	--	1	--	166	27	1	--	1	--
Vaccinated	141	137	1.83	(1.25 - 2.67)	1.49	(0.93 – 2.37)	215	63	1.80	(1.10-2.95)	1.56	(0.88-2.78)
GOLD 2 (N=2995)												
Unvaccinated	457	330	1	--	1	--	619	168	1	--	1	--
Vaccinated	658	622	1.31	(1.09 – 1.57)	1.11	(0.90 – 1.37)	962	318	1.22	(0.98-1.51)	1.00	(0.78-1.27)
GOLD 3 (N=1333)												
Unvaccinated	183	214	1	--	1	--	286	111	1	--	1	--
Vaccinated	318	425	1.14	(0.89 – 1.46)	0.92	(0.69 – 1.21)	508	235	1.19	(0.91-1.56)	1.00	(0.73-1.35)
GOLD 4 (N=183)												
Unvaccinated	34	25	1	--	1	--	44	15	1	--	1	--
Vaccinated	42	66	2.14	(1.12 – 4.07)	3.85	(1.49 – 9.93)	69	39	1.66	(0.82-3.36)	3.74	1.29-10.79

OR: crude odds ratio. ORa: odds ratio adjusted for age (continuous variable), gender, concomitant asthma diagnosis, smoking status (ordinal variable: non-smoker, former smoker, current smoker), number of moderate exacerbations the previous year, number of severe exacerbations the previous year, and the following comorbities: Heart failure, Atrial fibrillation, Cor pulmonale, Anxiety disorder, Osteoporosis, Allergic rhinitis, Gastroesophageal reflux disease, and Diabetes. COPD: chronic obstructive pulmonary disease. COPD obstruction severity: GOLD 1: mild; GOLD 2 moderate; GOLD 3: severe; GOLD 4 very severe.

Table S4. Crude and adjusted associations between "History of Influenza Vaccination" and risk of moderate COPD Exacerbations" in patients with confirmed COPD (FEV1/FVC < 0.7) by obstruction severity during the complete year and the epidemic period.

	2013 Frequent exacerbator phenotype					
	**No**	**Yes**				
	N=3100	*N*=1143	OR	(95% CI)	ORa	(95% CI)
Influenza Vaccination (2012-2013 Campaign) Total (N=4243)						
Unvaccinated	1213	386	1	--	1	--
Vaccinated	1887	757	1.26	(1.09–1.45)	1.03	(0.86–1.23)
GOLD 1 Mild (>=80%) (N=471)						
Unvaccinated	164	29	1	--	1	--
Vaccinated	217	61	1.59	(0.98–2.59)	0.95	(0.52–1.74)
GOLD 2 Moderate (50 to <80%) (N=2067)						
Unvaccinated	615	172	1	--	1	--
Vaccinated	972	308	1.13	(0.92–1.40)	0.91	(0.71–1.18)
GOLD 3 Severe (30 to <50%) (N=1140)						
Unvaccinated	280	117	1	--	1	--
Vaccinated	475	268	1.35	(1.04–1.76)	1.06	(0.77–1.45)
GOLD 4 Very Severe <30% (N=167)						
Unvaccinated	38	21	1	--	1	--
Vaccinated	59	49	1.50	(0.78–2.89)	3.44	(1.36–8.69)

OR: crude odds ratio. ORa: odds ratio adjusted for age (continuous variable), gender, concomitant asthma diagnosis, smoking status (ordinal variable: non-smoker, former smoker, current smoker), number of moderate exacerbations the previous year, number of severe exacerbations the previous year, and the following comorbities: Heart failure, Atrial fibrillation, Cor pulmonale, Anxiety disorder, Osteoporosis, Allergic rhinitis, Gastroesophageal reflux disease, and Diabetes. COPD: chronic obstructive pulmonary disease. COPD obstruction severity: GOLD 1: mild; GOLD 2 moderate; GOLD 3: severe; GOLD 4 very severe.
